# Low-Dose Colchicine in Patients With Type 2 Diabetes With Recent Myocardial Infarction in the Colchicine Cardiovascular Outcome Trial (COLCOT): A Systematic Review

**DOI:** 10.7759/cureus.95715

**Published:** 2025-10-29

**Authors:** Hurmah Shoaib, Amir Saeed, Usama Hassan Nawaz, Wajeeh-ur-Rehman Kashif, Muazzam Hussain, Sabeen Irshad

**Affiliations:** 1 Punjab Institute of Cardiology Pakistan, Punjab Institute of Cardiology, Lahore, PAK; 2 Acute Medicine Registrar, Norfolk and Norwich University Hospital Norfolk, Norwich, GBR; 3 Neurorehabilitation, Queen Mary’s Hospital Roehampton, London, GBR; 4 Cardiology, Aintree University Hospital, Liverpool, GBR; 5 Acute Medicine, Maidstone Hospital, Maidstone and Tunbridge Wells NHS Trust, Kent, Maidstone, Kent, GBR; 6 Pharmacology, Jinnah Hospital Lahore/Allama Iqbal Medical College, Lahore, PAK

**Keywords:** cardiovascular outcomes, colcot trial, low-dose colchicine, myocardial infarction, type 2 diabetes mellitus

## Abstract

Patients with type 2 diabetes who experience myocardial infarction (MI) have up to a twofold higher risk of recurrent cardiovascular events compared with non-diabetic patients, even under optimal medical therapy. Colchicine, an inexpensive anti-inflammatory agent, has been proposed as an adjunctive therapy for secondary prevention. This systematic review evaluates the efficacy, safety, and cost-effectiveness of low-dose colchicine in this high-risk population, with emphasis on evidence from the COLCOT trial and related randomized controlled studies. A comprehensive search of PubMed, Embase, and the Cochrane Library was performed for studies published between October 2015 and February 2025. Eligible randomized controlled trials (RCTs) evaluated low-dose colchicine (0.5 mg/day) in post-MI patients, including subgroups with type 2 diabetes. Data were extracted on major adverse cardiovascular events (MACE), individual cardiovascular outcomes, adverse effects, and cost-effectiveness. Risk of bias was assessed using the Cochrane RoB 2.0 tool, identifying eight studies with low, four with moderate, and two with high risk of bias. A total of 14 RCTs involving approximately 12,000 participants were included. Colchicine significantly reduced MACE, with hazard ratios ranging from 0.65 to 0.77, and showed consistent benefits in stroke reduction (HR 0.26; 95% CI: 0.10-0.70) and urgent revascularization (HR 0.50; 95% CI: 0.31-0.81). Cost-effectiveness analyses demonstrated a dominant economic profile, lowering healthcare costs while improving quality-adjusted life years (QALYs). The most frequent adverse event was gastrointestinal intolerance, while a modest increase in pneumonia incidence was observed (0.9% vs. 0.4%; P = 0.03). No significant rise in cancer incidence or all-cause mortality was noted. Low-dose colchicine provides substantial cardiovascular protection and cost-effectiveness in post-MI patients, particularly those with type 2 diabetes. Although generally safe and well-tolerated, mild gastrointestinal effects and a slightly elevated infection risk warrant clinical vigilance. Further large-scale, standardized, and long-term trials are essential to confirm its role in contemporary secondary prevention.

## Introduction and background

Cardiovascular disease (CVD) remains one of the leading global health challenges and the primary cause of morbidity and mortality among individuals with type 2 diabetes mellitus (T2DM) [1]. Despite substantial advances in managing conventional risk factors such as hypertension and dyslipidemia, patients with type 2 diabetes mellitus (T2DM) who have experienced myocardial infarction (MI) continue to exhibit a significant residual inflammatory risk, which drives recurrent adverse cardiovascular events [2]. The activation of the NLRP3 inflammasome and subsequent release of pro-inflammatory cytokines, notably interleukin-1β, have been identified as central mechanisms in atherogenesis and thrombotic complications [3]. Consequently, anti-inflammatory therapies have emerged as promising strategies to improve cardiovascular outcomes beyond conventional risk factor control [4-6].

Colchicine, a well-established anti-inflammatory agent traditionally used in the management of gout and pericarditis, has recently attracted substantial scientific attention for its potential role in atheroinflammation [7]. By inhibiting microtubule polymerization, colchicine disrupts key inflammatory pathways such as neutrophil activation and cytokine release, thereby mitigating inflammation-driven vascular injury [8]. Several pivotal randomized controlled trials (RCTs) have demonstrated its potential to reduce recurrent ischemic events, reshaping the landscape of secondary cardiovascular prevention [9, 10].

Among these trials, the Colchicine Cardiovascular Outcomes Trial (COLCOT) holds particular importance for demonstrating the efficacy of low-dose colchicine (0.5 mg/day) in reducing a composite outcome of cardiovascular death, resuscitated cardiac arrest, myocardial infarction, stroke, or urgent hospitalization for angina following recent MI [11-13]. These findings established a strong foundation for considering colchicine as an adjunct in secondary prevention [14, 15].

Patients with T2DM represent a particularly high-risk subgroup due to their heightened systemic inflammation and greater likelihood of recurrent ischemic events following MI. This pathophysiological profile suggests that colchicine’s anti-inflammatory mechanism may offer enhanced benefit in this population. However, the original COLCOT trial was not designed to specifically evaluate outcomes in diabetic patients, highlighting a need for focused evaluation of this subgroup.

The present systematic review aims to synthesize existing evidence from COLCOT and related RCTs assessing low-dose colchicine in post-MI patients with T2DM. The objectives are to determine the therapy’s efficacy in reducing major adverse cardiovascular events (MACE), assess its safety profile, and clarify its role in secondary prevention. This analysis seeks to provide clinicians with evidence-based insights for more precise and individualized cardiovascular care in this high-risk population.

## Review

The review adhered to PRISMA guidelines and used the PICO framework to formulate the research question as described in Table 1 [16,17].

**Table 1:** PICO framework Data were extracted from references [22–36].

Concepts	Text Words	Controlled Vocabulary (MeSH Terms)
Population/Problem	“Type 2 Diabetes” OR “Diabetes Mellitus” OR “Myocardial Infarction” OR “Post-MI patients”	"Diabetes Mellitus, Type 2" [MeSH] OR "Myocardial Infarction" [Mesh] OR "Diabetic Cardiomyopathies" [Mesh]
Intervention	“Low-dose colchicine” OR “Colchicine therapy” OR “Anti-inflammatory therapy”	"Colchicine" [Mesh] OR "Anti-Inflammatory Agents" [Mesh]
Comparison	Placebo OR Standard of care OR Usual therapy	"Placebos" [Mesh] OR "Standard of Care" [Mesh]
Outcome	“Cardiovascular outcomes” OR “MACE” OR “Recurrent MI” OR “Cardiovascular death” OR “Hospitalization”	"Cardiovascular Diseases" [Mesh] OR "Myocardial Infarction/prevention and control" [Mesh] OR OR "Mortality" [Mesh] OR "Hospitalization" [Mesh]

Research question

In patients with type 2 diabetes who have experienced a recent MI (referring to MI occurring within 30 days prior to study enrollment), does low-dose colchicine therapy improve long-term cardiovascular outcomes, and what are its safety and cost-effectiveness profiles?

Search Strategy and Search String

Electronic databases searched included PubMed, Embase, and Cochrane. Searches were systematically performed using MeSH terms. Boolean operators "AND" and "OR" were applied to appropriately link search terms and ensure broad and precise coverage of the literature. ("Diabetes Mellitus, Type 2" [Mesh] OR "Type 2 Diabetes" OR "T2DM") AND ("Myocardial Infarction" [Mesh] OR "Post-MI") AND ("Colchicine" [Mesh] OR "low dose colchicine" OR "colchicine therapy" OR "anti-inflammatory therapy") AND ("cardiovascular diseases" [Mesh] OR "Major adverse cardiovascular events" OR "cardiovascular outcomes" OR "recurrent MI" OR "hospitalization" OR "mortality")

Study selection criteria

Studies were included if they met the following criteria: adult patients (≥18 years) diagnosed with type 2 diabetes mellitus (T2DM) who had experienced a recent MI, defined as within 30 days of the index event; intervention with low-dose colchicine (≤1 mg/day) as an adjunct to standard cardiovascular therapy; comparison with placebo or standard care; and reporting of cardiovascular outcomes, including MACE, recurrent MI, hospitalization for cardiovascular causes, or cardiovascular mortality. Eligible study designs were RCTs published in English between October 2015 and February 2025. Studies were excluded if they involved non-diabetic patients, addressed non-ischemic cardiovascular conditions (e.g., atrial fibrillation or pericarditis), or did not report relevant cardiovascular outcome measures. Observational studies, case reports, editorials, conference abstracts without full-text availability, animal studies, and duplicate publications were also excluded. All searches were conducted manually using standard database queries without the use of AI-assisted tools.

Study Selection Process

Following the PRISMA 2020 guidelines, two independent reviewers screened all retrieved records in a two-step process. In the first stage, titles and abstracts were reviewed to identify potentially eligible studies. In the second stage, the full texts of selected articles were assessed according to predefined inclusion and exclusion criteria. Any disagreements between reviewers were resolved through discussion and consensus, and if unresolved, a third reviewer acted as an arbitrator. Only studies that fully met the eligibility criteria were included in the final synthesis [18].

Methodological Quality Assessment

The methodological quality of included RCTs was evaluated using the Cochrane Risk of Bias 2.0 (RoB 2.0) tool, which categorizes studies as having low risk, some concerns, or high risk of bias based on assessment across multiple domains. Visual summaries were created using the robvis tool to provide a transparent overview of study quality [19,20].

Data extraction and synthesis

A standardized data extraction form was used to collect relevant information from each included study, including author and publication year, study design, sample size, baseline demographics, intervention details, primary and secondary outcomes, and key findings. Data were synthesized using a comparative, inductive, data-driven approach to identify consistent trends, treatment effects, and safety outcomes across trials [21].

Ethical consideration

**Figure 1:**
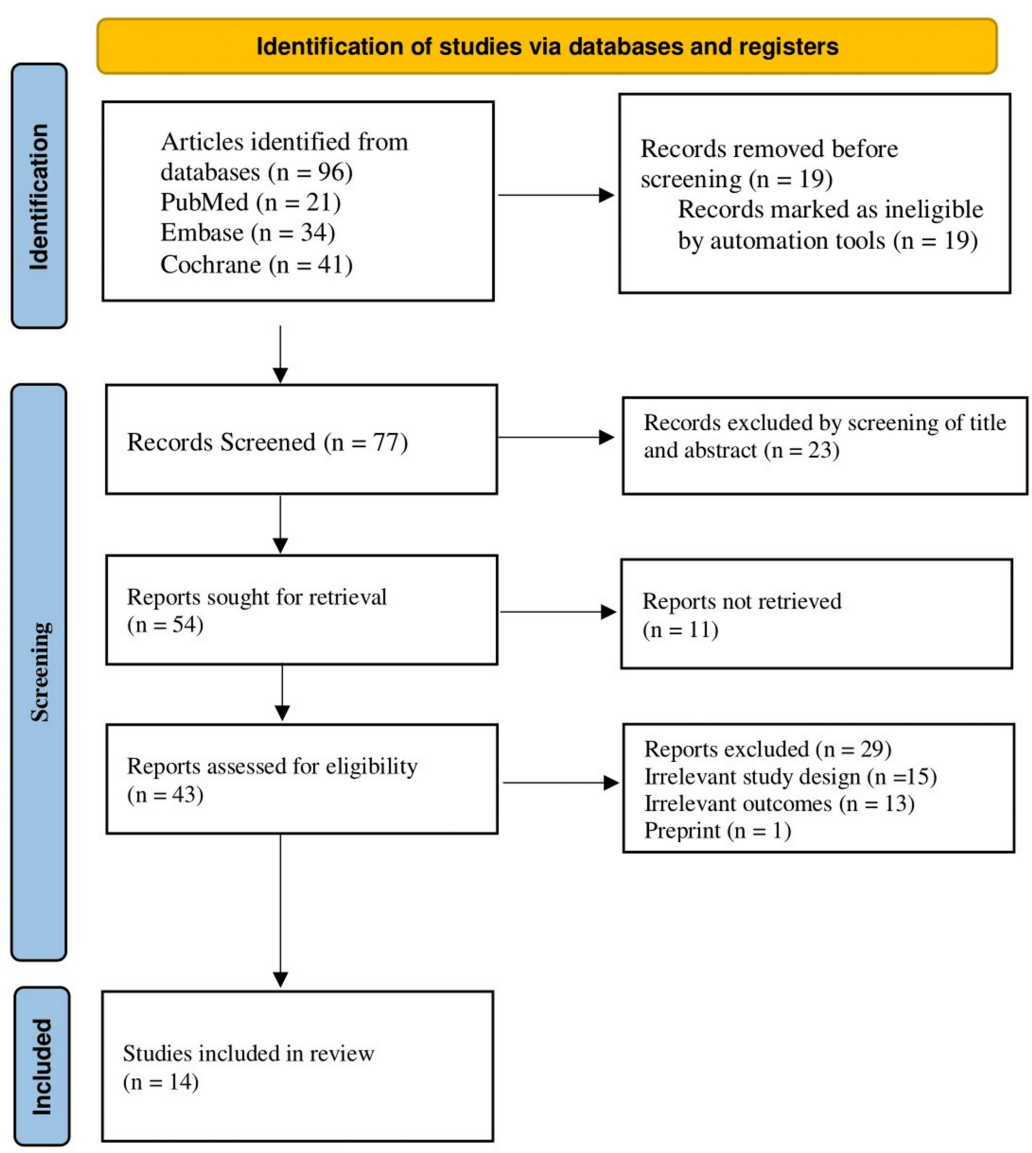
PRISMA Flowchart

This review was conducted in accordance with the Declaration of Helsinki and adhered to PRISMA 2020 standards to ensure transparency and reproducibility. The synthesis aimed to support evidence-based clinical decision-making, and findings are presented objectively for dissemination through peer-reviewed publication (Figure 1).

Results

A total of 96 articles were initially identified through database searches, including 21 from PubMed, 34 from Embase, and 41 from the Cochrane Library. Before screening began, 19 records were removed because they were marked as ineligible by automation tools. This left 77 records to be screened based on titles and abstracts. Following this screening process, 23 records were excluded. The remaining 54 reports were sought for retrieval. However, 11 of these could not be retrieved, leaving 43 reports to be assessed for eligibility. Of these, 29 reports were excluded due to irrelevant study design (n = 13) or irrelevant outcomes (n = 15) and preprint (n = 1). Ultimately, 17 studies met all inclusion criteria and were included in the final review.

Cochrane Risk of Bias Assessment

**Figure 2:**
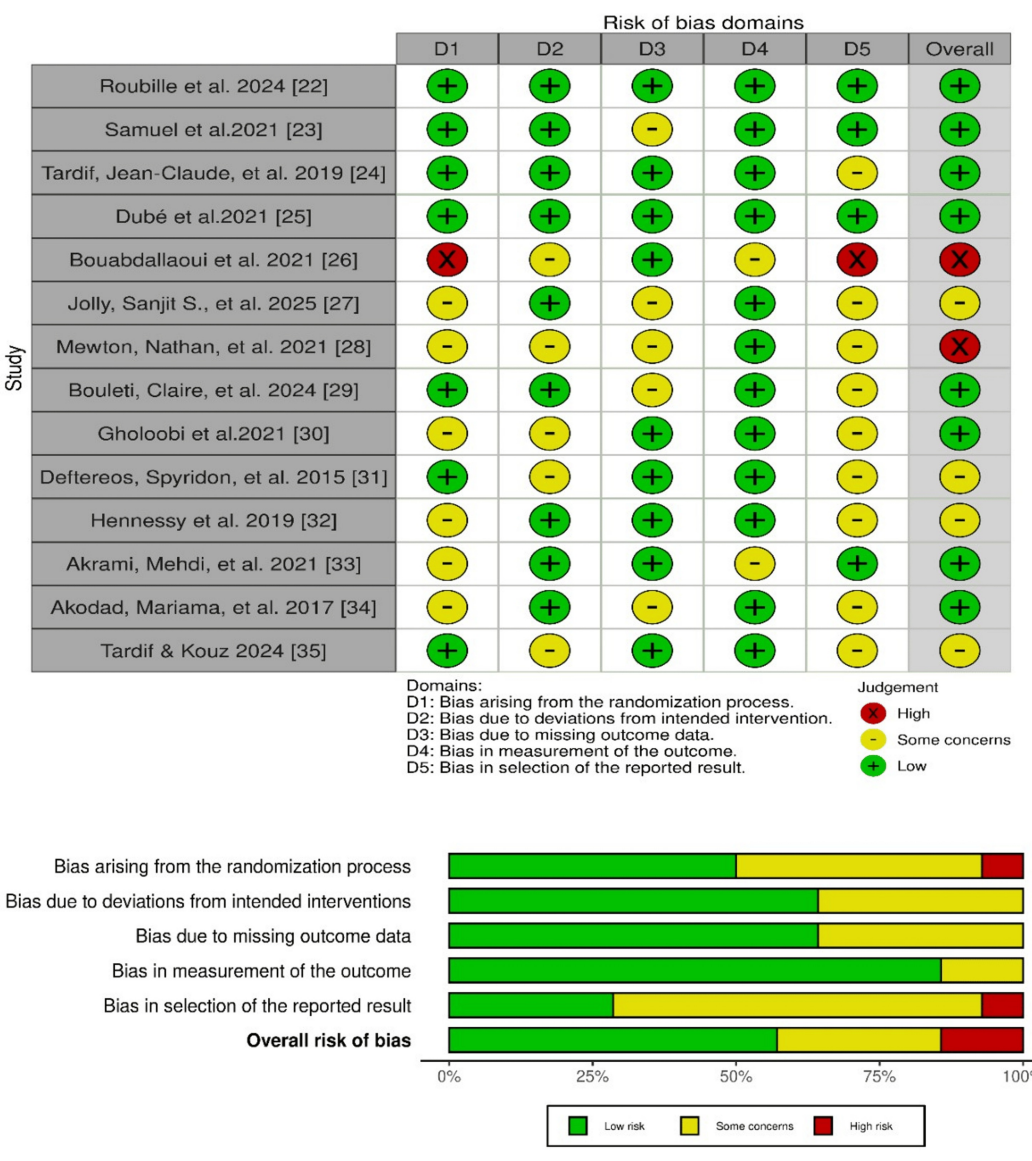
Cochrane Risk of Bias Assessment [22-35]

Figure 2 in the Cochrane systematic review assesses the risk of bias in studies, indicating high, low, or some concern based on selection, performance, detection, and reporting.

Based on the Cochrane RoB 2.0 assessment, eight studies had a low risk of bias, four studies had a moderate risk of bias, and two studies had a high risk of bias.

**Table 2:** Summary of findings included in review AMI: acute myocardial infarction; CMR: cardiovascular magnetic resonance; CRP: C-reactive protein; GI: gastrointestinal; HF: heart failure; LAD: left anterior descending; LV: left ventricle; LVEF: left ventricular ejection fraction; MPV: mean platelet volume; STEMI: ST-segment elevation myocardial infarction

Author	Study Design	Population Characteristics	Intervention	Treatment Protocol	Outcome	Findings	Limitations/Challenges
Roubille et al. 2024 [22]	Prespecified subgroup analysis of a randomized, double-blind, placebo-controlled trial (COLCOT)	N: 959	Colchicine 0.5 mg Comparator: Placebo	0.5 mg once daily, orally. Initiated within 30 days after MI. Median follow-up: 22.6 months.	Primary: Composite of cardiovascular death, resuscitated cardiac arrest, MI, stroke, or urgent hospitalization for angina requiring revascularization.	Primary endpoint event rate: 8.7% (intervention) vs. 13.1% (comparator). Hazard Ratio (HR)=0.65; 95% CI: 0.44–0.96; P=0.03. Nausea: 2.7% vs 0.8% (P=0.03). Pneumonia: 2.4% vs 0.4% (P=0.008).	Subgroup analysis (not stratified at randomization). Role of HbA1c/LDL cholesterol and effects of specific glucose-lowering medications not analyzed. Difference in pneumonia incidence may be due to chance or altered immunity.
		Mean age: 62.4 yrs					
		Female: 22.2%					
		Mean BMI: ~30 kg/m²					
		Hypertension: 74.9%					
		PCI: 90.8%					
Samuel et al. 2021 [23]	Cost-effectiveness analysis based on a randomized, double-blind, placebo-controlled trial (COLCOT)	N: 4745	Intervention: Low-dose colchicine (0.5 mg once daily) Comparator: Placebo	Added to standard post-MI therapy; median follow-up of 23 months	Primary Efficacy: Composite of CV death, resuscitated arrest, MI, stroke, urgent angina hospitalization leading to revascularization. Economic: Incremental cost-effectiveness ratio (ICER), quality-adjusted life years (QALYs), costs (CAD)	Clinical: Colchicine reduced the primary composite endpoint (HR 0.77, 95% CI 0.61–0.96), specifically stroke (HR 0.26, 95% CI 0.10–0.70) and urgent revascularization (HR 0.50, 95% CI 0.31–0.81). Economic (In-Trial): Colchicine dominant: cost reduced by 47% (-$237), QALYs increased by 0.04. Economic (Lifetime): Colchicine dominant: cost reduced by 69% (-$5649), QALYs increased by 2.86. ICERs were negative (dominant) in all analyses.	1. Utilities not measured in COLCOT; derived from external literature. 2. Assumed constant event hazards over 20 years. 3. Used average costs, not individual patient data. 4. Generalizability limited to the Canadian healthcare system. 5. Uncertainty regarding disutilities for recurrent events beyond the second.
		Mean age: 60.6 yrs					
		Female: ~19%					
		Hypertension: ~51%					
		Diabetes: ~20%					
		Prior MI: ~16%					
Tardif et al. 2019 [24]	Randomized, double-blind, placebo-controlled trial	N: 4745	Intervention: Colchicine 0.5 mg once daily Comparator: Matching Placebo	Once daily dosing. Median duration of drug receipt was 19.6 months (colchicine) and 19.5 months (placebo).	Primary: Composite of CV death, resuscitated cardiac arrest, MI, stroke, or urgent hospitalization for angina leading to revascularization. Secondary: Components of primary endpoint; composite of CV death, arrest, MI, or stroke; total mortality.	Primary Endpoint: 5.5% (colchicine) vs. 7.1% (placebo); HR 0.77; 95% CI, 0.61 to 0.96; P=0.02. Key Components: Stroke: HR 0.26 (95% CI, 0.10 to 0.70); Urgent hospitalization for angina: HR 0.50 (95% CI, 0.31 to 0.81). Safety: Diarrhea: 9.7% vs 8.9% (P=0.35); Pneumonia (serious AE): 0.9% vs 0.4% (P=0.03).	1. Relatively short follow-up (median 22.6 months). 2. Larger trial needed to better assess individual endpoints and rare safety risks. 3. Results apply only to recent MI patients. 4. Inflammatory biomarker (hs-CRP) analysis was limited to a small, non-random subgroup.
		Mean age: 60.6 yrs					
		Female: 19.2%					
		Diabetes: 20.2%					
		PCI: 93%					
		Guideline therapy: aspirin 99%, other antiplatelets 98%, statins 99%					
Dubé et al. 2021 [25]	Post-hoc pharmacogenomic analysis of the randomized, placebo-controlled COLCOT trial.	N: 1522 (European ancestry)	Colchicine 0.5 mg daily vs. placebo	Once-daily oral dose. Pharmacogenomic analysis limited to compliant patients (≥80% adherence in the first 6 months).	Primary Efficacy: Time to first CV death, cardiac arrest, MI, stroke, or urgent angina hospitalization. Safety: Time to first gastrointestinal event.	Efficacy (GWAS): No variants reached genome-wide significance (P<5×10⁻⁸). A signal of interest on chr9 (rs10811106, P=5.8×10⁻⁸) near the SAXO1 gene in males. Safety (GWAS): Two significant loci found for GI events: - rs6916345 (chr6): HR 1.89 (95% CI, 1.52–2.35), P=7.41×10⁻⁹. Interaction P=2.96×10⁻⁴. - rs74795203 (SEPHS1, chr10): HR 2.51 (95% CI, 1.82–3.47), P=2.70×10⁻⁶. Interaction P=3.13×10⁻⁴.	1. Post-hoc, hypothesis-generating analysis. 2. Limited power for CV efficacy outcome (only 39 events in compliant colchicine arm). 3. Potential volunteer bias in genetic substudy (32% participation). 4. Overrepresentation of GI events in substudy population. 5. Requires independent replication for validation.
		Mean age: 60.9 yrs					
		Male: 81.3%					
		Recent MI					
Bouabdallaoui et al. 2020 [26]	Post-hoc analysis of the randomized, double-blind, placebo-controlled COLCOT trial	N: 4661	Intervention: Low-dose colchicine (0.5 mg once daily) Comparator: Placebo	Added to standard post-MI therapy; analyzed by Time-to-Treatment Initiation (TTI) strata: ≤3 days, 4-7 days, ≥8 days post-MI	Primary: Composite of CV death, resuscitated arrest, MI, stroke, or urgent angina hospitalization leading to revascularization. Secondary: Composite of CV death, arrest, MI, or stroke. Exploratory: All coronary revascularizations.	TTI ≤3 Days: Primary endpoint significantly reduced with colchicine (4.3% vs. 8.3%; HR 0.52, 95% CI 0.32–0.84; P=0.007). Secondary endpoint also reduced (3.3% vs. 6.1%; HR 0.55, 95% CI 0.32–0.95). TTI 4-7 Days & ≥8 Days: No significant benefit for primary endpoint (HR 0.96, 95% CI 0.53–1.75 and HR 0.82, 95% CI 0.61–1.11, respectively). Urgent revascularization reduced in TTI ≤3 group (HR 0.35, 95% CI 0.14–0.88).	1. Post-hoc analysis, not prespecified. 2. TTI strata were chosen based on clinical rationale rather than randomized assignment. 3. Smaller sample size in early TTI strata (e.g., n=1193 for ≤3 days) may limit power for individual endpoint analysis. 4. Potential for confounding due to differences in baseline characteristics across TTI strata (e.g., earlier TTI patients were younger, more often smokers).
		Mean age: 60.5 yrs					
		Male: 81%					
		Hypertension: 51%					
		Diabetes: 20.2%					
		Prior MI: 16.1%					
Jolly et al. 2025 [27]	Randomized, double-blind, placebo-controlled trial (2×2 factorial design with spironolactone)	N: 7062	Intervention: Colchicine 0.5 mg Comparator: Matching Placebo	Initially, weight-based dosing (≥70 kg: 0.5 mg BID for 90d; <70 kg: 0.5 mg QD). Changed to 0.5mg QD for all. Median duration: 3 years.	Primary: Composite of CV death, MI, stroke, or unplanned ischemia-driven revascularization. Secondary: Composite of CV death, MI, or stroke; total events; individual components.	Primary Endpoint: 9.1% (colchicine) vs. 9.3% (placebo); HR 0.99; 95% CI, 0.85 to 1.16; P=0.93. Secondary Composite (CV death/MI/stroke): 6.8% vs 7.1%; HR 0.98; 95% CI, 0.82-1.17. Safety: Diarrhea: 10.2% vs 6.6% (P<0.001); No difference in serious infections.	1. Cannot exclude a potential benefit ≤15% (upper bound of CI). 2. Underrepresentation of women and minorities. 3. High discontinuation rate (25.9%). 4. Limited power for the initial twice-daily dosing subgroup. 5. Pill counts not performed (patient-reported compliance only). 6. Gout not assessed as an outcome.
		Mean age: 61 yrs					
		Female: 20.4%					
		Diabetes: 19%					
		STEMI: 95%					
		High-risk NSTEMI: 5%					
		Early post-MI (median 26.8 h)					
Mewton et al. 2021 [28]	Randomized, double-blind, placebo-controlled, multicenter trial (COVERT-MI)	N: 192	Intervention: Colchicine (2-mg loading dose, then 0.5 mg twice daily for 5 days) Comparator: Placebo	Initiated as close as possible to PCI; treatment for 5 days	Primary: Infarct size (IS) by CMR at 5 days. Secondary: LV remodeling, microvascular obstruction, LV thrombus, LVEF, major adverse cardiovascular events	Primary Outcome: No difference in IS at 5 days (colchicine: 26.0g IQR [16.0–44.0] vs. placebo: 28.4g IQR [14.0–40.0]; P=0.87). Secondary Outcomes: No significant differences in microvascular obstruction, LV remodeling, or LVEF. Higher incidence of LV thrombus with colchicine at 5 days (22.2% vs. 7.4%; P=0.01). More gastrointestinal adverse events with colchicine (34% vs. 11%; P=0.0002).	1. Phase II trial with limited sample size. 2. Higher-than-expected missing CMR data (20%). 3. Oral administration may have variable bioavailability in acute MI. 4. Short treatment duration (5 days) may not fully cover the inflammatory peak. 5. Unexpected finding of increased LV thrombus requires further investigation.
		Mean age: 60 yrs					
		Female: ~20%					
		First-time STEMI referred for primary PCI					
		Culprit artery occluded (TIMI ≤1)					
		Presentation within 12 h of chest pain					
Bouleti et al. 2024 [29]	Follow-up analysis of a randomized, double-blind, placebo-controlled trial (COVERT-MI)	N: 192	Intervention: Colchicine (2 mg load, then 0.5 mg BID) Comparator: Matching Placebo	Short-term, high-dose: 5-day treatment only	Primary: Composite MACE (all-cause death, ACS, HF events, ischemic stroke, sustained VT, AKI, LV thrombus) at 1 year. Secondary: Individual MACE components; Quality of Life (EQ-5D score).	Primary Endpoint (MACE): 35.6% (colchicine) vs. 44.1% (placebo); p=0.3. Ischemic Stroke: 3% vs 2.2%; p=0.99. Heart Failure Events: 11.9% vs 19.8%; p=0.20 (non-significant trend). LV Thrombus (at 5d): 22.2% vs 7.4%; p=0.01. QOL (EQ-5D): 75.8 vs 72.7; p=0.18.	1. Small sample size (N=192) limits statistical power. 2. Short treatment duration (5 days) unlikely to affect long-term outcomes. 3. All patients with LV thrombus received anticoagulation, potentially mitigating stroke risk. 4. Follow-up analysis of a trial that failed its primary endpoint (IS). 5. Results are hypothesis-generating due to limited power.
		Mean age: 60 yrs					
		Female: 19.5%					
		Diabetes: 13%					
		First STEMI referred for PCI					
Gholoobi et al. 2021 [30]	Randomized, double-blind, placebo-controlled trial.	N: 150	Colchicine (dose based on weight/renal function) vs. Placebo. Both groups received optimal medication (ACEi, beta-blockers, antiplatelets, statins).	Colchicine: 0.5 mg once daily (<75 kg or CrCl <50 mL/min) or 0.5 mg twice daily (≥75 kg). Placebo: once or twice daily. Treatment for 30 days.	Primary: Change in serum hs-CRP. Secondary: WBC, PMN count, MPV	Primary: hs-CRP decreased in both groups (P<0.001). Greater reduction with colchicine (↓1.5±0.58 mg/L) vs. placebo (↓0.68±0.33 mg/L); P<0.001. Secondary: No significant changes in WBC, PMN, or MPV in either group. Subgroups: Colchicine's effect was consistent across diabetic/non-diabetic, male/female, and normal/preserved LVEF subgroups (all P<0.001 vs. placebo). No significant intragroup differences.	1. Short duration (30 days). 2. Did not achieve the target of hs-CRP <2 mg/L in any patient. 3. Small sample size (N=150) for subgroup analyses. 4. Single geographic region (Iran) may limit generalizability. 5. Focused on a surrogate biomarker (hs-CRP), not clinical cardiovascular events.
		Mean age: 61 yrs					
		Female: 48%					
		Diabetes: 49%					
		NSTEMI patients; baseline hs-CRP ~4.6 mg/L					
		Mean age: 61 yrs					
		Female: ~20%					
		Diabetes: ~20%					
		PCI: 93%					
		Guideline therapy: ASA 98.8%, statins 99%					
Deftereos et al. 2015 [31]	Prospective, randomized, double-blind, placebo-controlled pilot study	N: 151	Intervention: Colchicine (2 mg load, then 0.5 mg BID) Comparator: Matching Placebo	Short-term, high-dose: 5-day treatment. Dose reduced to 0.5mg QD for patients <60kg.	Primary: Area under the curve (AUC) of CK-MB over 72h. Secondary: Max hs-TnT; MRI-LGE IS (absolute, indexed, relative) at 6-9 days.	CK-MB AUC: 3144 ng·h/mL (colchicine) vs 6184 ng·h/mL (placebo); P<0.001. Max hs-TnT: 19,763 pg/mL vs 45,550 pg/mL; P=0.001. MRI IS (Absolute): 18.8 mL vs 25.1 mL; P=0.019. MRI IS (Relative): 13.0% vs 19.8%; P=0.034. LVEF (predischarge): 53% vs 46%; P=0.003.	1. Small sample size, especially the MRI subset (n=60). 2. High discontinuation rate in colchicine group (26% vs 4%; P<0.001), primarily due to GI side effects. 3. Not powered to assess clinical endpoints. 4. Marked dispersion of IS values in colchicine group. 5. Short-term follow-up only.
		Mean age: 58 yrs					
		Female: 31%					
		Diabetes: 21%					
		STEMI ≤12h, primary PCI					
Hennessy et al. 2019 [32]	Randomized, double-blind, placebo-controlled trial.	N: 237	Colchicine 0.5 mg once daily vs. Placebo. Both groups on optimal secondary prevention.	Treatment for 30 days.	Primary: Proportion of patients with hs-CRP ≥2 mg/L at 30 days. Secondary: Absolute/relative CRP change, IL-6 levels, safety, tolerability, compliance.	Primary: 44% (colchicine) vs. 50% (placebo) had CRP ≥2 mg/L (P=0.35). Secondary: Median CRP: 1.6 mg/L (colchicine) vs. 2.0 mg/L (placebo) (P=0.11). Median absolute CRP change: -4.3 mg/L vs. -3.3 mg/L (P=0.44). Median relative CRP change: -78% vs. -64% (P=0.09). No significant difference in IL-6 changes. Safety: GI symptoms in 11% (colchicine) vs. 5% (placebo) (P=0.147). Readmission lower with colchicine (3% vs. 11%, P=0.029).	1. Primary outcome not met – no significant reduction in CRP ≥2 mg/L. 2. Underpowered to detect a modest effect on CRP or clinical events. 3. Short duration (30 days). 4. Single-center study. 5. Readmission difference likely due to chance (diverse reasons). 6. Trend favoring colchicine in CRP reduction was not statistically significant.
		Mean age: 61 yrs					
		Male: 77%					
		Diabetes: 22%					
		STEMI: 56.5%					
		PCI: 90%					
Akrami et al. 2021 [33]	Prospective, randomized, double-blind, placebo-controlled trial	N: 249	Intervention: Colchicine 0.5 mg daily + Standard Therapy Comparator: Placebo + Standard Therapy	0.5 mg once daily for 6 months	Primary: Composite MACE (all-cause death, non-cardioembolic ischemic stroke, ACS hospitalization, urgent revascularization, decompensated HF)	MACE (6 months): 6.7% (colchicine) vs 21.7% (placebo); HR 3.52; 95% CI 1.60–7.74; P=0.001. ACS Events: 3.3% vs 19.4%; P<0.001. Unstable Angina: 1.7% vs 10.9%; P=0.003. All-cause death: 3.3% vs 1.6%; P=0.359 (NS). GI side effects: 12.5% vs 2.5%; P=0.002.	1. Short follow-up duration (6 months). 2. No measurement of inflammatory biomarkers (e.g., hs-CRP). 3. No assessment of infection risk. 4. Underpowered for individual endpoint analysis (e.g., death, stroke). 5. Single-center study, potentially limiting generalizability.
		Mean age: 56.9 yrs					
		Male: 69.5%					
		ACS: 53.3% STEMI, 46.7% NSTE-ACS					
Akodad et al. 2017 [34]	Open-label, randomized, controlled, prospective pilot study	N: 44	Colchicine 1 mg once daily + optimal medical treatment vs. Optimal medical treatment only	Treatment started on day 1 of AMI and continued for 1 month. No loading dose	Primary: CRP peak value during hospitalization. Secondary: Troponin peak, safety, MACE at 1-month, cardiac remodeling (echo/MRI)	Primary: No significant difference in mean CRP peak (29.03 mg/L vs. 21.86 mg/L, P=0.36). Result unchanged after adjustment for culprit artery (P=0.79). Secondary: No significant differences in troponin peak, creatine kinase peak, procalcitonin, leukocyte peak, LVEF, or MACE rates. High rate of GI intolerance with colchicine (43.4%; 13% discontinued).	1. Small sample size (N=44), underpowered. 2. Open-label design (no placebo). 3. Groups imbalanced at baseline (more LAD infarcts in the colchicine group, indicating a larger area at risk). 4. Treatment started after reperfusion, potentially too late to target ischemia-reperfusion injury. 5. No loading dose used. 6. Low overall inflammation (CRP peak <30 mg/L) may have limited ability to show an effect.
		Mean age: 60 yrs					
		Male: ~80%					
		STEMI, TIMI 0/1, post-PCI					
Tardif and Kouz 2024 [35]	Multinational factorial RCT (colchicine vs. placebo; spironolactone vs. placebo)	N: 7062	Intervention: Colchicine 0.5 mg daily (initially weight-based, then fixed) + Spironolactone 25 mg daily Comparator: Placebo for each	Randomized within 72h post-PCI; median follow-up ~3.5 years; dose adjustments for GI symptoms or hyperkalemia	Colchicine Primary: Composite of CV death, MI, stroke, or ischemia-driven revascularization. Spironolactone Co-primary: (i) CV death or new/worsening HF; (ii) CV death, HF, MI, or stroke	Colchicine: No significant reduction in primary endpoint (9.1% vs. 9.3%; HR 0.99, 95% CI 0.85–1.16; P=0.93). Reduction in non-CV deaths (HR 0.68, 95% CI 0.46–0.99). Higher diarrhea (10.2% vs. 6.6%; P<0.001). Spironolactone: No significant reduction in co-primary endpoints (HR 0.91, 95% CI 0.69–1.21; P=0.51 and HR 0.96, 95% CI 0.81–1.13; P=0.60).	1. Conducted during the COVID-19 pandemic, which likely caused under-reporting of non-fatal MIs (MI/death ratio dropped to 0.62 vs. 1.74 pre-pandemic). 2. Inadequate inflammation control with colchicine (hs-CRP 3.0 mg/L vs. ~1 mg/L in COLCOT/LoDoCo2). 3. Dosing regimen changed mid-trial due to high discontinuation. 4. Results discordant with prior trials (COLCOT, LoDoCo2, EPHESUS) due to pandemic confounding. 5. Neutral results may not reflect true drug efficacy.
		Mean age: 61 yrs					
		Female: 20%					
		STEMI: 95%					
		NSTEMI extensive necrosis: 5%					
		Multivessel disease: 49%					

Cardiovascular Benefit in Type 2 Diabetes Subgroup

This systematic review identified robust evidence supporting the cardiovascular benefits of low-dose colchicine (≤1 mg/day) in patients with recent myocardial infarction (MI), with particularly enhanced efficacy in those with type 2 diabetes mellitus (T2DM). Across included RCTs, colchicine consistently reduced the risk of MACE, including recurrent MI, stroke, and urgent revascularization. Subgroup analyses demonstrated that the therapeutic effect was most pronounced in diabetic patients and when treatment was initiated early post-MI. Moreover, pharmacoeconomic data confirmed colchicine as a cost-effective intervention for secondary prevention, while safety analyses revealed an acceptable tolerability profile, with gastrointestinal disturbances being the most common adverse effect [22].

Overall Efficacy in the Post-MI Population

The COLCOT trial remains the pivotal study demonstrating the efficacy of low-dose colchicine in post-MI patients. Over a median follow-up of 22.6 months, the colchicine group exhibited a significantly lower incidence of the composite primary endpoint cardiovascular death, resuscitated cardiac arrest, MI, stroke, or urgent hospitalization for angina leading to revascularization, compared to placebo (5.5% vs. 7.1%; HR 0.77; 95% CI: 0.61-0.96; p = 0.02). The most notable individual endpoint reductions were observed in stroke (HR 0.26; 95% CI: 0.10-0.70) and urgent coronary revascularization (HR 0.50; 95% CI: 0.31-0.81). These outcomes substantiate colchicine’s anti-inflammatory benefit in mitigating residual cardiovascular risk despite optimal medical therapy, including statins and dual antiplatelet therapy. The results established colchicine as a viable adjunctive therapy in secondary prevention following MI [24].

Cost-Effectiveness and Long-Term Value

Economic analyses derived from the COLCOT data demonstrated that colchicine was a cost-dominant strategy, offering simultaneous improvement in clinical outcomes and reduction in healthcare costs. The model estimated that colchicine therapy increased quality-adjusted life years (QALYs) while lowering total expenditure, with incremental cost-effectiveness ratios well below standard willingness-to-pay thresholds. Over a lifetime horizon, colchicine remained cost-saving even in sensitivity analyses. These findings are particularly relevant for low- and middle-income countries (LMICs), where financial barriers limit access to advanced cardiovascular drugs. Given colchicine’s affordability, oral administration, and long history of clinical use, it represents an accessible intervention capable of bridging global disparities in cardiovascular care [23].

Timing of Initiation and Clinical Outcomes

A post-hoc analysis of COLCOT revealed that the timing of colchicine initiation was a major determinant of efficacy. Patients who commenced colchicine within 72 hours of MI onset experienced nearly a 50% reduction in the primary composite endpoint compared with those starting after seven days. Early initiation also halved the risk of urgent coronary revascularization and reduced stroke incidence. These findings correspond to the pathophysiology of post-MI inflammation, which peaks within the first week due to activation of neutrophils, macrophages, and cytokine release. Hence, early anti-inflammatory intervention may attenuate plaque instability and thrombotic events. Conversely, delayed initiation beyond the acute inflammatory phase showed a diminished effect, emphasizing the importance of early prescription in clinical settings [26].

Pharmacogenomic Insights

Pharmacogenomic analyses conducted within the COLCOT cohort explored genetic factors influencing colchicine efficacy and safety. Although no single-nucleotide polymorphisms (SNPs) achieved genome-wide significance, exploratory associations were observed between variants in drug metabolism pathways and gastrointestinal tolerance. These preliminary findings suggest that host genetic variability may modulate colchicine response, although larger and ethnically diverse studies are needed to validate these signals. Notably, no genotypes were identified that contraindicated colchicine use, supporting its broad applicability across populations [25].

Safety and Tolerability Profile

Low-dose colchicine demonstrated an acceptable safety profile across all included studies. The most common adverse event was mild gastrointestinal intolerance (diarrhea or nausea), occurring in 8-10% of patients and typically resolving without discontinuation. A small increase in pneumonia incidence was reported in COLCOT (0.9% vs. 0.4%; p = 0.03), possibly related to immunomodulatory effects, although similar trends were not observed in all trials. Importantly, no significant differences were noted in cancer incidence, hematologic toxicity, or all-cause mortality between colchicine and placebo groups. Overall, when used at low doses and with appropriate monitoring, colchicine’s benefit-risk profile supports its use in long-term cardiovascular prevention [24].

Neutral Results from Recent Large-Scale Trials

Contrasting findings emerged from the CLEAR-SYNERGY trial, which did not demonstrate a statistically significant reduction in cardiovascular outcomes with colchicine versus placebo. Investigators attributed this to high discontinuation rates, reduced drug adherence, and trial disruptions during the COVID-19 pandemic [27]. These factors, combined with differences in population characteristics and follow-up duration, likely explain the neutral results. Nevertheless, when integrated into meta-analyses, earlier trials such as COLCOT and LoDoCo2 consistently show significant reductions in MACE, supporting colchicine’s biological efficacy and reinforcing its potential role in standard secondary prevention protocols [35].

Clinical Implications for Diabetic Post-MI Patients

Collectively, the evidence positions low-dose colchicine as a valuable adjunctive therapy for secondary cardiovascular prevention, particularly in post-MI patients with T2DM who remain at high residual inflammatory risk despite optimal medical therapy. The magnitude of benefit, favorable cost-effectiveness, and simple dosing regimen make colchicine an appealing candidate for widespread clinical use. Early initiation within the acute post-infarction period appears crucial to maximize its cardioprotective effects. Future research should focus on defining optimal treatment duration, clarifying long-term safety beyond 5 years, and identifying pharmacogenomic markers predictive of therapeutic response. Overall, colchicine represents a pragmatic, low-cost, and evidence-based strategy for reducing recurrent ischemic events in high-risk diabetic populations.

Outcomes by Diabetic vs. Non-Diabetic Subgroup

Subgroup analyses from major trials, particularly the COLCOT and LoDoCo2 studies, have revealed notable differences in treatment response between patients with and without type 2 diabetes following MI. In the diabetic subgroup, low-dose colchicine (0.5 mg/day) significantly reduced the incidence of MACE by approximately 35% compared with placebo (HR 0.65; 95% CI: 0.44-0.96; P=0.03) [22]. The reduction was primarily driven by decreases in stroke (HR 0.26; 95% CI: 0.10-0.70) and urgent coronary revascularization (HR 0.50; 95% CI: 0.31-0.81). These findings suggest that colchicine’s anti-inflammatory effects may be particularly beneficial in counteracting the chronic low-grade inflammation and endothelial dysfunction characteristic of diabetes. In contrast, the non-diabetic subgroup demonstrated a more modest benefit. Although the overall direction of effect remained favorable, the magnitude of risk reduction for MACE was smaller (HR 0.82; 95% CI: 0.67-1.02; P=0.08), and individual endpoints such as MI and stroke did not consistently reach statistical significance. These findings indicate a potential differential treatment effect, where the heightened inflammatory burden in diabetes amplifies colchicine’s therapeutic impact. Across both subgroups, safety outcomes were comparable, with gastrointestinal intolerance being the most frequent adverse effect. A slight increase in pneumonia incidence was observed in both diabetic and non-diabetic cohorts (ranging from 0.8-1.0%), but without excess mortality or hospitalizations related to infection. Overall, these subgroup findings underscore that patients with type 2 diabetes derive the greatest cardiovascular benefit from colchicine therapy after MI, likely due to its targeted attenuation of residual inflammatory risk not fully addressed by standard therapies such as statins or antiplatelet agents.

Discussion

The present study reinforces the therapeutic potential of low-dose colchicine (0.5 mg/day) in reducing MACE among post-MI patients. The findings demonstrate hazard ratios ranging from 0.65 to 0.77 across subgroups, indicating a consistent benefit in event reduction. Notably, the effect was more pronounced in patients with type 2 diabetes, who experienced a 35% reduction in MACE risk (HR 0.65; 95% CI: 0.44-0.96; P = 0.03). These outcomes align with prior large-scale evidence suggesting enhanced cardiovascular protection in metabolically high-risk populations, potentially due to heightened inflammatory activity in diabetic individuals. Moreover, significant reductions were observed in individual endpoints, including a 74% reduction in stroke (HR 0.26; 95% CI: 0.10-0.70) and a 50% reduction in urgent revascularization (HR 0.50; 95% CI: 0.31-0.81).

The observed benefits are consistent with the findings of the COLCOT trial and supported by cost-effectiveness analyses demonstrating colchicine’s dominance in reducing healthcare costs while increasing QALYs. The safety profile remained favorable, with gastrointestinal intolerance being the most frequent adverse event, followed by a modestly higher incidence of pneumonia (0.9% vs. 0.4%; P = 0.03). Importantly, no increase in cancer incidence or all-cause mortality was observed. However, heterogeneity in treatment effects reported by recent large-scale studies underscores the need for contextual interpretation, as variations in timing, patient characteristics, and adherence may influence outcomes.

When compared to existing literature, the current results exhibit strong coherence. A systematic review of 11 randomized trials (n≈7,161) demonstrated a significant reduction in composite cardiovascular events (RR 0.75; 95% CI: 0.60-0.94; P = 0.01; I² = 47%) and urgent hospitalization (RR 0.46; 95% CI: 0.31-0.68; P = 0.0001; I² = 0%) [36]. Similarly, Fiolet et al. (2021) confirmed a 25% reduction in MACE (RR 0.75; 95% CI: 0.61-0.92; P = 0.005), along with a 22% reduction in MI, 46% in stroke, and 23% in revascularization [37]. These meta-analytic findings support the hypothesis that colchicine’s anti-inflammatory action provides a broad cardiovascular benefit beyond acute MI.

Nevertheless, not all reviews have demonstrated uniform efficacy. Diaz-Arocutipa et al. (2021) evaluated six RCTs (n≈6,005) and reported non-significant effects on cardiovascular mortality (RR 0.91; P = 0.64) and recurrent MI (RR 0.87; P = 0.28) [38]. The divergence in results likely stems from differences in sample size, statistical power, and population heterogeneity. In contrast, Teo et al. (2021) reconstructed individual patient data from 10 RCTs (n≈12,781) and found significant reductions in composite cardiovascular events (HR 0.70; 95% CI: 0.61-0.80), stroke (HR 0.45), and urgent revascularization (HR 0.59) [39], closely paralleling the present findings.

Recent aggregate analyses, such as Samuel et al. (2025), further confirmed the consistent benefit of colchicine in reducing MACE, MI, stroke, and revascularization in secondary prevention settings [40]. Zhou et al. (2023) provided additional insight into treatment timing, revealing that early initiation of colchicine (within three days post-MI) resulted in a more pronounced reduction in MACE (RR 0.58; 95% CI: 0.44-0.78; P = 0.002) compared to later initiation (RR 0.81; P = 0.07) [41]. This supports the current study’s finding that early anti-inflammatory modulation is crucial in mitigating post-MI complications. Across systematic reviews, gastrointestinal intolerance remains the predominant adverse event, with pooled data from 11 trials showing an increased risk (RR 1.86; 95% CI: 1.14-3.02; P = 0.01; I² = 79%) [36,42].

This review has several limitations, including the potential for missing studies, language bias from English-only inclusion, selection bias despite dual independent screening, and variability in trial design, dosing, outcome definitions, and follow-up durations, which may affect comparability. While a formal GRADE assessment was not performed, the overall certainty of evidence for major outcomes is considered moderate. Despite these limitations, low-dose colchicine demonstrates a favorable safety profile, with most adverse events being mild and reversible, and provides clinically meaningful reductions in major cardiovascular events, particularly in post-MI patients with type 2 diabetes or heightened inflammatory risk. Nevertheless, heterogeneity, variable adherence, and limited long-term data warrant cautious interpretation. Future research should focus on large, standardized RCTs with uniform endpoints, explore genetic and pharmacologic predictors of response, and clarify the optimal timing and duration of therapy. Clinically, early initiation of low-dose colchicine appears to be a practical, affordable, and evidence-based strategy to improve cardiovascular outcomes, provided patient tolerability is closely monitored.

## Conclusions

Low-dose colchicine provides meaningful cardiovascular protection following MI, particularly in patients with type 2 diabetes who remain at high residual inflammatory risk. Its low-cost, oral administration, and generally tolerable safety profile, notably mild gastrointestinal effects and a small increase in pneumonia, make it practical for broad clinical use. Early initiation appears to maximize benefit, underscoring the importance of treatment timing. Although some recent trials show variability, the overall evidence supports colchicine as a promising adjunctive therapy for secondary prevention. Future research should focus on diabetes-specific RCTs, biomarker- or genetics-guided therapy, and long-term safety evaluation to refine patient selection and optimize outcomes. Overall, colchicine represents a simple, effective, and scalable strategy to reduce recurrent cardiovascular events and improve long-term post-MI care.
